# Consumer perceptions of community pharmacists' involvement in antimicrobial stewardship: A quantitative study

**DOI:** 10.1016/j.rcsop.2023.100281

**Published:** 2023-05-20

**Authors:** Kathryn Lim, Elaine Lum, Lisa Nissen, Alex Broom, Holly Seale

**Affiliations:** a School of Population Health, University of New South Wales, Australia; b Duke-NUS Medical School, National University of Singapore, Singapore; c Centre for the Business and Economics of Health, The University of Queensland, Australia; d School of Social and Political Sciences, The University of Sydney, Australia

**Keywords:** Antimicrobial stewardship, Antimicrobial resistance, Pharmacy, Primary care

## Abstract

Background Community pharmacist involvement in antimicrobial stewardship (AMS) within primary care is underutilised. Despite this view being consistently held across the pharmacy sector's policy, academic and professional spheres, there is limited understanding of how this positioning aligns with consumers' perceptions and expectations. Objective To explore participants' experience using antibiotics and their engagement with pharmacists to support their use. Methods Online survey of Australian adults recruited via Dynata's research panel in November 2022. Questions were organised into three sections: 1) understanding the participant's use of antibiotics, including their information needs; 2) exploring engagement with pharmacists on a cold and flu enquiry using a vignette question; and 3) demographic information. Results Doctors (42.0%), pharmacists (29.8%) and the internet including general searches (14.3%) were the top three sources for antibiotic information. Information about side effects and anticipated time to effect were more broadly sought from pharmacists than what was provided. Over 50% of respondents indicated alignment between the best practice example of a pharmacist providing cold and flu management advice with their own experience. 17% of respondents indicated that they would seek doctor's advice when considering cold and flu management options compared to 10% seeking pharmacist's advice. No statistically significant results between age groups or gender were observed. Conclusion Better visibility of community pharmacists' involvement in managing minor ailments in primary care, including more explicit linkage of pharmacist-administered vaccination services as an AMS strategy can support optimal antimicrobial use.

## 1 Background

Focused efforts to increase public awareness and understanding about antimicrobial resistance (AMR) are a mainstay of global action to reduce AMR.^1^ This is connected to the premise that increasing awareness and understanding of AMR as a global health threat, creates a sense of shared ownership across sectors and members of society to tackle this issue through collective effort.^1^^,^^2^ Messaging in AMR public awareness campaigns seek to influence people's AMR knowledge, attitudes and beliefs, as research has suggested that these factors may drive inappropriate antimicrobial use.^3^ Broadly, these key messages have focused on providing alerts on the scale of the issue, building an understanding of the efficacy of antimicrobial agents, and highlighted what is considered appropriate use.^4^ These key messages are often simultaneously broadcasted to health professionals and consumers during AMR public awareness campaigns, to facilitate a complementary and reinforcing dynamic^4^ between these two groups when engaging about AMR.

Primary care professionals, such as general practitioners and pharmacists, are key target audiences of AMR public awareness campaigns. This reflects the increasing global focus on involving primary care as part of the whole-of-system effort to reduce AMR^5^; recognising that this clinical setting is a key access point for accessing healthcare and health information, and inappropriate antimicrobial prescribing or dispensing in the community is another contributing avenue to AMR.^5^

However, qualitative research by Lum and colleagues (2017) exploring Australian consumers' perspectives, attitudes and behaviours about AMR highlighted a gap between consumers' expectations and the reality of information provided by general practitioners and pharmacists about AMR.^6^ According to this study, participants indicated that these two health professional groups did not always voluntarily meet consumers' AMR information needs, which led to consumers seeking alternative information sources to fill this gap.^6^ Further, research has also suggested that information provided to consumers is not clear on what action they can take to reduce AMR.^6^^,^^7^

Pharmacists are recognised as key contributors in promoting the optimal use of antimicrobials.^8^ Much has been made of the perceived “untapped potential”^9^ that exists for pharmacists, particularly in the community setting, in contributing to optimising antimicrobial use. Harnessing pharmacists' specialist knowledge in medicines,^9^ inherent interactions with consumers and prescribers when managing antimicrobial prescriptions^6^^,^^10^ and a commitment to supporting the quality use of medicines have been promoted as key reasons to support their involvement in reducing AMR. These attributes has been promoted by peak pharmacy organisations through their position statements^11^ and professional publications.^12^ This view was echoed in results from a qualitative study of key informants across clinical practice, program and/or program development or research settings with the Australian community pharmacy sector.^13^

Consumer knowledge about the role of community pharmacists has been shown to influence expectations of what the profession can and should provide by way of healthcare services.^14^ This equally extends to community pharmacists' contribution to mitigating AMR – where despite consistency across the sector's policy, academic and professional views on a clear role for community pharmacy involvement in antimicrobial stewardship (AMS),^11^^,^^13^ there is limited understanding of how this positioning aligns with consumers' perceptions and expectations. Therefore, this study explored consumers' experience of seeking information from, and engaging with community pharmacists on using antimicrobials as a starting point to address this research gap.

## 2 Methods

A cross-sectional online survey of Australian adults was conducted between 18 November 2022 and 24 November 2022. The online survey was programmed using the Qualtrics platform, with participants recruited through the commercial survey platform, Dynata. Dynata (https://www.dynata.com/) maintains a global panel of voluntary market research participants who receive points as incentives for participation, which panel members can then redeem for cash or prizes. Dynata distributed the survey link to a random sample of their panel members residing in Australia. Panel members who were eligible to participate needed to be 18 years or over, able to read and understand English and reside in Australia. The Participant Information Statement was made available for download, and starting the survey was taken as eligible participants providing informed consent. Sample size calculations indicated a minimum of 385 completed surveys were required for a 95% confidence interval and 5% margin of error, based on an estimate of 21,000,000 Australians aged 18 years or over from Australian Bureau of Statistics data at the time of the survey.^15^ The Human Research Ethics Committee at the University of New South Wales granted approval (HC#22065) for this study.

The survey (Supplementary File 1) used closed questions to explore participants' experience using antibiotics and their engagement with pharmacists to support their use. For the purposes of this survey, the more familiar term ‘antibiotics’ was used over ‘antimicrobials’. The survey questions were organised into three sections: 1) understanding the participant's use of antibiotics, including their information needs; 2) exploring engagement with pharmacists on a cold and flu enquiry using a vignette question; and 3) demographic information. The survey was piloted with an initial sample of 25 respondents for face and content validity.

Questions exploring participants' use of antibiotics and their information needs were informed by research findings from Lum and colleagues (2017)^6^ of consumer expectations of information provided regarding antibiotics, Hawke and colleagues' (2015)^16^ analysis of antibiotic related enquiries from consumers to an Australian medicines helpline, and a checklist-intervention piloted as part of a UK feasibility study exploring a community pharmacy AMS intervention.^17^ These questions explored the self-reported gap between the expectations and reality of information received regarding antibiotic prescription as highlighted in previous research.

A vignette question was used to explore participant's interactions with pharmacists about a cold and flu enquiry. The vignette was adapted from a case study published by the UK's Royal Pharmaceutical Society which described how community pharmacists could support cold and flu interactions.^18^ As a ‘best practice’ example, this vignette described providing information about AMR, appropriate use of antimicrobials, appropriate treatment options including seeking further advice, and highlighting AMS supporting activities such as vaccinations as key domains. The questions explored consumers' self-management approach to upper respiratory tract infections (URTI), and were informed by previous research exploring consumers' perceptions and rationale for seeking pharmacy-based services for minor ailments,^19^ and consumer expectations relating to consultation and antibiotics for URTI.^20^

Demographic information, including age, gender and ethnicity was collected. The ethnicity questions were informed by Diversity Council Australia research,^21^ with response options and reporting outputs aligned to relevant Australian Bureau of Statistics (ABS) Standards.^22^, 23., 24

Data were collated in Microsoft Excel and imported to SPSS (IBM SPSS Statistics for Windows, Version 26.0. Armonk, NY, USA) for further analysis. Descriptive statistics, including frequency percentages and means were used to present the survey results. Cross-tabulations and chi-square or Fisher's exact test were used to examine if antibiotic information needs varied based on the demographic variables of age or gender. Age and gender were also tested using a one-way ANOVA (analysis of variance) to examine if these variables had a significant effect on engaging with a pharmacist on a cold and flu enquiry as explored through a Likert scale. A Tukey post hoc test was to be conducted should a significant difference be shown. Across all analyses, a value of *p* < 0.05 was considered statistically significant.

## 3 Results

Seven hundred and forty-seven people opened the survey. Among eligible participants, 404 completed the survey. Participants that did not respond to the age screener question (*n* = 7) or had data quality issues (*n* = 1) were excluded from analysis, resulting in a final sample of 397. The characteristics of the study's participants are shown in Table 1. The mean age of respondents was 47 years (SD = 17), with 50.1% identifying as female. Over 80% indicated that English was their only spoken language.

**Table 1:** Survey respondent demographics (*n* = 397).

Characteristics and categories	Frequency (n)	Percentage (%)
Gender		
Male	177	44.6
Female	199	50.1
Non-binary or gender diverse/Prefer not to say	21	5.3
Age groups		
18–24	45	11.3
25–34	69	17.4
35–44	87	21.9
45–54	71	17.9
55–64	55	13.9
65 and older	70	17.6
Location		
New South Wales	105	26.4
Victoria	90	22.7
Queensland	71	17.9
South Australia	44	11.1
Western Australia	44	11.1
Tasmania	15	3.8
Northern Territory	1	0.3
Australian Capital Territory	9	2.3
Unknown	18	4.5
Indigenous status		
Aboriginal and/or Torres Strait Islander	18	4.5
Non-Indigenous	359	90.4
Not stated/inadequately described	20	5.0
Cultural background and ethnicity^a^		
Oceanian	163	32.9
North-West European	264	53.3
Southern and Eastern European	25	5.1
North African and Middle Eastern	6	1.2
South-East Asian	5	1.0
North-East Asian	20	4.0
Southern and Central Asian	11	2.2
Peoples of the Americas	1	0.2
Sub-Saharan Africa	0	0.0
Languages spoken^a^		
English	321	83.6
Other language	62	16.2

a Multiple response options allowed.

Understanding respondents' antibiotic related information needs was limited to those having self-reported antibiotic use in the last 12 months. Over half met these criteria (*n* = 259, 65.2%), with 52% identifying as female (*n* = 135). Table 2 shows respondents' self-reported access points for antibiotic information. Doctors (*n* = 221, 42.0%), pharmacists (*n* = 157, 29.8%) and the internet including general searches (*n* = 75, 14.3%) were the top three sources for antibiotic information. Of these three sources of information, 39.5% (n = 157) indicated that a doctor would be their first point to access information about antibiotics, followed by pharmacists (*n* = 46, 11.6%). Results from a chi-square test of independence revealed that there were no statistically significant differences between the first point of access for antibiotic information by age groups (*X*^*2*^*(35, N* *=* *222) =* *38.0, p* *=* *0.3)* or by gender (*X*^*2*^*(10, N* *=* *214) = 4.8, p* *=* *0.9)*.

**Table 2:** Self-reported access points for antibiotic information.

	Access points for antibiotic information^a^		First point of access for antibiotic information	
	Frequency (n=)	Percentage (%)	Count (n=)	Percentage (%)
Doctor	221	42.0%	157	70.7%
Pharmacist	157	29.8%	46	20.7%
Family or friends	31	5.9%	5	2.3%
Internet, including a general search	75	14.3%	11	5.0%
Publications, such as pamphlets	23	4.4%	1	0.5%
Social media	17	3.2%	2	0.9%
Other	2	0.4%	–	–
Total	526	100.0%	222	100.0%

a Multiple response options allowed in response to question “Where would you go to seek information about antibiotics?”

**Fig. 1:**
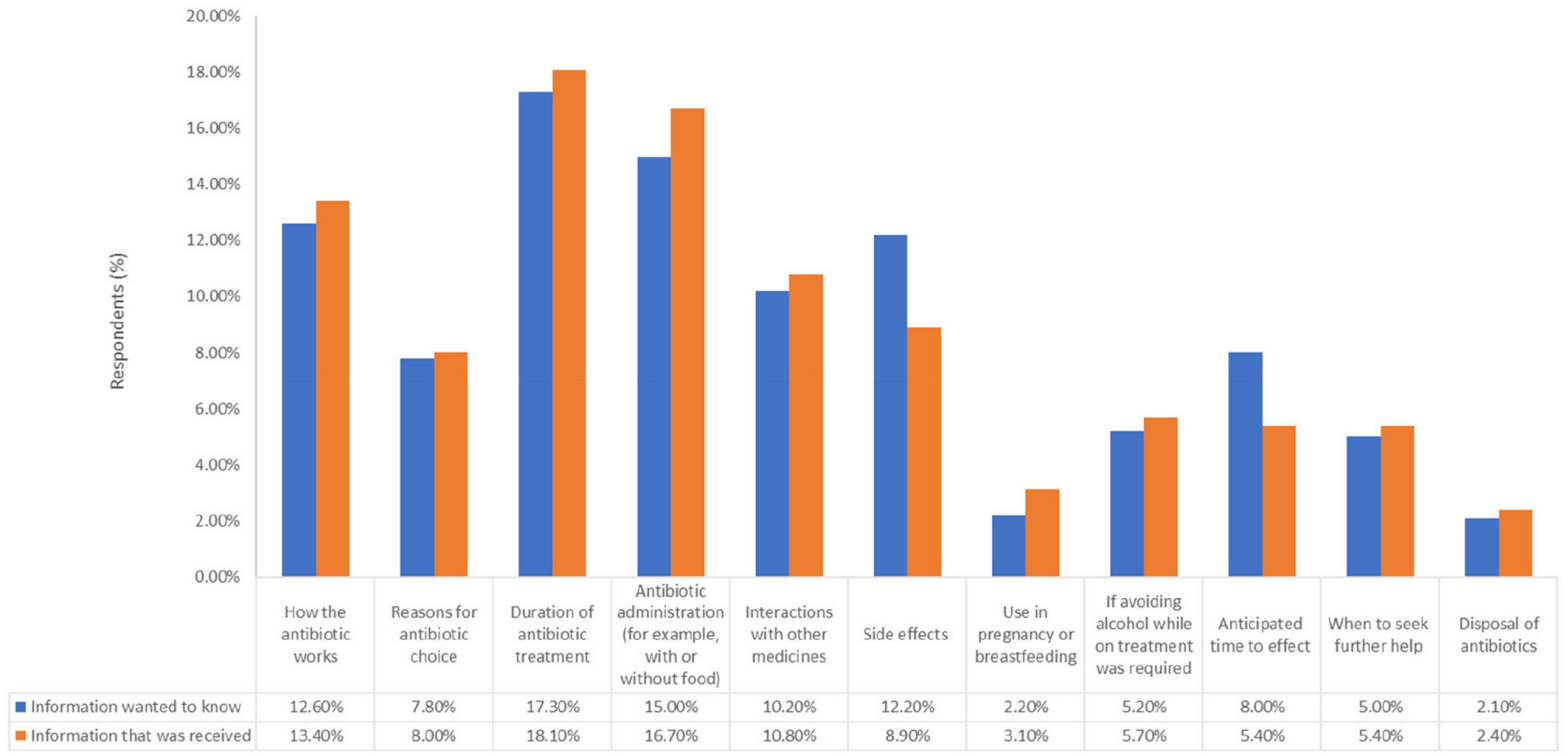
Percentage of responses to “What information did you want to know” (*n* = 898), and “What information did you receive” (*n* = 777), multiple responses allowed.

Out of 250 responses, filling a new prescription provided by a doctor was the major source (n = 221, 88.4%) for respondents in obtaining their most recent antibiotic. Of the 240 respondents who indicated having filled an antibiotic prescription – where the prescription was either new (*n* = 221) or previously issued (*n* = 19) – information provided by pharmacists matched what they wanted to know across most information categories (Fig. 1). However, there was a difference observed in the percentage of respondents between the information they wanted compared to what they received about side effects (12.20% (*n* = 110) vs. 8.90% (*n* = 69)) and the anticipated time to effect (8.00% (*n* = 72) vs 5.40% (*n* = 42)). This result was the same across age groups, but was not statistically significant. Few indicated that they either had no questions (*n* = 18, 2.00%) or that they did not receive any information (*n* = 14, 1.80%).

Table 3 shows respondents' self-reported practices when having a common cold, with “asking a pharmacist for advice” accounting for 10.1% (*n* = 93) of responses. The top three reasons for doing so were because the pharmacist would be able to tell them if they needed to see a doctor (*n* = 50, 19.5%), they did not consider their symptoms serious enough for a doctor's visit (*n* = 49,19.1%), or it was easier to speak to a pharmacist for advice (*n* = 39, 15.2%).

**Table 3:** Self-reported practices for a common cold (*n* = 922, multi-choice response).

	Frequency (n)	Percentage (%)
Consult a doctor, including having a telehealth appointment	157	17.0%
Ask a pharmacist for advice	93	10.1%
Take medicines that I already have at home	139	15.1%
Have extra rest	198	21.5%
Take home remedies, such as sipping hot liquids	119	12.9%
Take vitamins, such as Vitamin C	124	13.4%
Ask a family member or friend for advice	26	2.8%
Search the internet to find advice on how to feel better	30	3.3%
Take leftover or unused antibiotics	11	1.2%
Visit the emergency department	9	1.0%
Other	16	1.7%

**Fig. 2:**
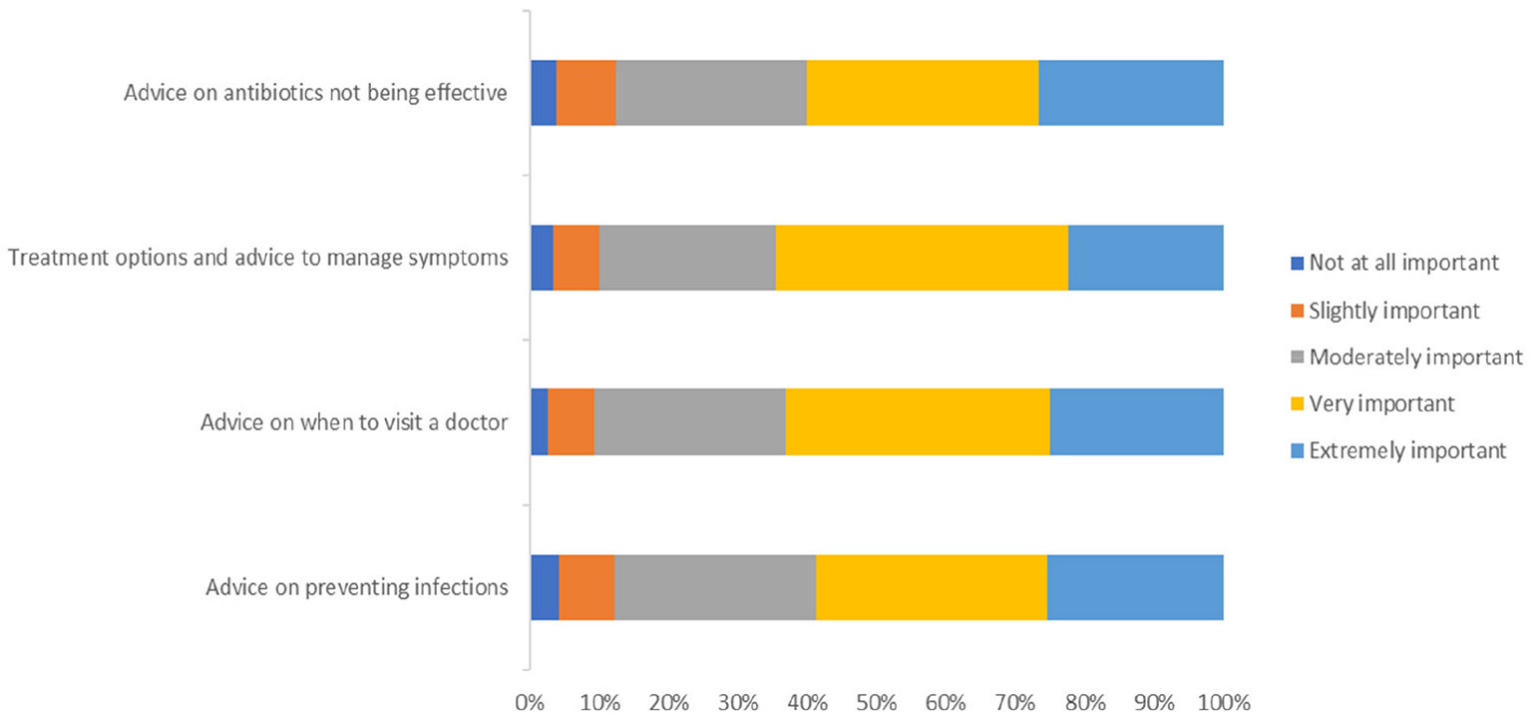
Level of agreement with the importance of the topics of information presented in the vignette (Likert scale: 5 = not at all important, 1 = very important).

Mean scores (±SD) across all information topics covered in the vignette question were considered ‘moderately important’ (Fig. 2) - advice on antibiotics not being effective (3.7 ± 1.1), treatment options to manage symptoms (3.7 ± 1.0), advice on when to visit a doctor (3.8 ± 1.0), and advice on preventing infections (3.7 ± 1.0). One way ANOVA results reported no statistically significant difference among age group means. Over half (*n* = 68, 58.6%) of the 114 respondents who self-reported visiting a pharmacy in the previous six months for a cold and flu enquiry “agreed” (based on a 5-point Likert scale) that the vignette accurately described their experience. This advice was sought for themselves by most respondents (*n* = 91, 64.1%). The difference in means was not statistically significant between genders (F = 2.0, *p* = 0.2) or age groups (F = 1.6, p = 0.2).

## 4 Discussion

Our findings suggest that the public regard pharmacists as one of their key sources for antibiotic related information, and expectations on what information pharmacists will provide about antibiotics are broadly met. However, seeking pharmacists' advice on minor ailments did not appear at the forefront of options considered by our study's respondents, despite increasing positioning of community pharmacy involvement in delivering minor ailments services by governments globally, in seeking to maximise the efficiency and effectiveness of their health systems.^25^

Doctors and pharmacists continue to occupy positions as key information brokers about antibiotics as highlighted in our findings,^26^ despite the rise in availability and accessibility of health information enabled by the internet. Knowledge about AMR has been highlighted as potentially influencing antibiotic supply practices by health professionals,^27^ and evidence of knowledge variability particularly in how AMR develops and spreads was highlighted in a 2019 quantitative study exploring European health professionals' antibiotic knowledge, attitudes and behaviours.^28^

Therefore, continued efforts to support primary care professionals to maintain up-to-date knowledge about antibiotics is critical as they play both a reinforcing role for information disseminated in public health campaigns, and are able to actively engage individuals in shared decision making which can support optimal antibiotic use.^29^ This includes moving beyond correcting misinformation about antibiotics to discussing what individuals can do to reduce AMR,^7^^,^^30^ such as advising on how to prevent infections. Further, this includes a need to be sensitive to cultural influences and practices, in a considered effort to support the whole community to engage in AMS.^31^ Our findings also suggest that pharmacists could consider focusing efforts on delivering information about side effects and anticipated time to effect; with this type of information sought more broadly from consumers when seeking medicines-related information.^14^

Previous research suggests that community pharmacists' breadth of influence to promote AMS behaviour is greater when fulfilling a ‘triage’ function.^13^ This has been reflected in community pharmacists being increasingly positioned as supporting increased optimal use of antimicrobials with regards to minor ailments,^9^^,^^32^ such as upper respiratory tract infections (URTIs) - one of the most common conditions treated in primary care managed with antibiotic use.^33^ Research suggests that this is achieved through community pharmacists supporting reduced referrals to GPs for self-limiting, viral based conditions – minimising the likelihood of unwarranted antibiotic prescribing and harnessing the opportunity to provide AMS related information to a consumer.^28^^,^^32^

Our findings suggest that the value of pharmacists' ability to triage is recognised by the public in supporting their determination of whether doctor's advice should be sought; reflecting the rhetoric promoted by the profession's peak organisations.^11^ However, respondents indicated that they would seek doctor's advice ahead of a pharmacist's when considering options to manage a cold and flu. This may indicate a narrow public appreciation of the breadth of pharmacists' skills and professional services offerings,^34^ despite the evolving identity of the profession from “dispenser” to “health care provider”.^35^^,^^36^

In Australia, this may reflect the lack of distinction in pharmacists' management of minor ailments as a discreet service in comparison to international models which have codified community pharmacy minor ailments schemes such as management of colds and flus.^37^ Our findings suggest that many respondents experienced interactions on cold and flu enquiries that reflected the best practice example, independent of a formalised service in Australia. However, understanding the breadth of pharmacists' skills appears to be connected to personal experience. Rodgers and colleagues (2016)^38^ compared pharmacist and public views on their experiences of community pharmacy medicines related services in England, and concluded that engaging with one pharmacist-led service may increase awareness and therefore potential use of other services.

Formalising a community pharmacy minor ailments service may be one approach to support improved community pharmacy involvement in AMS. This would act as an organising framework to harness key enablers for primary care AMS such as building general practice and community pharmacy collaboration and articulating roles and responsibilities^13^^,^^39^ which leverage the complementary, but distinct roles of each profession in tackling AMR.

However, there is an immediate opportunity to better promote the linkages between existing pharmacy professional services to broader efforts tackling AMR. In particular, this could include leveraging the global rise in authorisation of vaccine administration by pharmacist^40^ by actively promoting how this service supports the reduction of AMR development,^41^ and is a tangible action that can be taken by individuals as part of their contribution to AMS. There is an opportunity for future research to explore how the connection between infection prevention and control (IPC) measures such as immunisation and AMS is currently promoted by community pharmacists, and public receptivity on this message as part of community pharmacists' broader role in supporting public health.^42^

Our study has several limitations. Participant recruitment was limited to a consumer panel which may not be truly representative of the general population, and the use of incentives for participation and non-response from panellists who were invited to complete the survey but did not engage, may have introduced response bias.^43^ However, the consumer panel approach has been used in other research studies. Further, the self-reported nature of the data are subject to recall biases. The survey was administered online, which may have unintentionally excluded those without access to the internet. The survey was also only conducted in English, which may have resulted in exclusion of non-English speakers.

Though Indigenous status and ethnicity data were collected, it was not incorporated in the analysis due to the complexity associated with the multiple response options. Indigenous rural and remote primary health care plays an important role in supporting communities to address the rising rates of AMR in their communities.^44^ Further, ethnicity and culture has been described as shaping migrant communities' understandings, practices and experiences of AMR.^31^ There is an opportunity for further research to explore how pharmacists can specifically support AMS within these respective contexts.

## 5 Conclusion

Community pharmacists are regarded by the public as one of the key information brokers to source antibiotic related information. However, better visibility of the capability of community pharmacists to manage minor ailments in primary care and explicit linkage of vaccination services as an AMS strategy are needed to better support optimal antimicrobial use.

## Declaration of Competing Interest

None.

## Appendix A Supplementary data

Survey - Consumer perceptions of community pharmacists’ involvement in antimicrobial stewardship Image 1
