# Supplementary material for: Consumer perceptions of community pharmacists' involvement in antimicrobial stewardship: A quantitative study
Source: Explor Res Clin Soc Pharm. 2023 May 20;10:100281. doi: 10.1016/j.rcsop.2023.100281 (PMC10236209; doi:10.1016/j.rcsop.2023.100281)
Supplement: Supplementary file 1 — Survey - Consumer perceptions of community pharmacists’ involvement in antimicrobial stewardship [file mmc1.docx]

**Survey – Consumer perceptions of community pharmacists’ involvement in antimicrobial stewardship**

| **#** | **Question** |
| --- | --- |
| **SECTION 1 – ANTIBIOTIC USE AND INFORMATION NEEDS**  This section will ask you about your use of antibiotics and information you’d like about antibiotics. | |
| **1.1** | **Have you taken an antibiotic in the last 12 months?**   - Yes - No – *Skip to Section 2* |
| **1.2** | **Where would you go to seek information about antibiotics? You can select as many boxes as needed.**   - Doctor - Pharmacist - Family or friends - Internet, including a general search - Publications, such as pamphlets - Social media, such as Facebook, YouTube, Twitter - Other, please specify |
| **1.3** | **From the information sources you selected, which of these would be your first point to access information about antibiotics?**   - *Options carry forward from respondents’ answers in Question 1.2 – Select one option only* |
| **1.4** | **For the most recent antibiotic that you took, how did you get it?**   - Filled a new prescription that my doctor gave me – *Go to Question 1.5* - Filled a previous prescription that I had so I did not have to go to a doctor – *Go to Question 1.5* - Took leftover or unused antibiotics that I had at home – *Go to Section 2* - Took someone else’s leftover or unused antibiotics – *Go to Section 2* - Other, specify_____________ |
|  | **The next two questions will ask you about the antibiotic prescription you filled at the pharmacy** |
| **1.5** | **Please complete the following sentence by selecting from the following options. You can select as many boxes as needed.**  **The information or advice I wanted to hear about the antibiotic was…?**   - How does the antibiotic work - Why was this antibiotic chosen - How long I would need to take the antibiotic for - How should I take the antibiotic – for example, with or without food - Whether the antibiotic will interact with other medicines I am taking - What side effects I could have because of the antibiotic - Whether the antibiotic is safe for use during pregnancy or breastfeeding - Whether I need to avoid alcohol while on my antibiotic - How long it would take for me to feel better - When I should ask for more help with my infection - What to do with any antibiotics I have not used - Nothing, I had no questions - Other, please specify |
| **1.6** | **Please complete the following sentence by selecting from the following options. You can select as many boxes as needed.**  **The information or advice I was given about my antibiotic was…?**   - How does the antibiotic work - Why was this antibiotic chosen - How long I would need to take the antibiotic for - How should I take the antibiotic – for example, with or without food - Whether the antibiotic will interact with other medicines I am taking - What side effects I could have because of the antibiotic - Whether the antibiotic is safe for use during pregnancy or breastfeeding - Whether I need to avoid alcohol while on my antibiotic - How long it would take for me to feel better - When I should ask for more help with my infection - What to do with any antibiotics I have not used - I didn’t receive any information - Other, please specify |
|  | **SECTION 2 – SCENARIO BASED**  **This section will ask you questions based on a scenario about having a cold.** |
| **2.1** | **Imagine for the past two days, you have had a runny nose, cough and are sneezing occasionally. After a negative COVID-19 test result, which of the following actions would you be most likely to take? Please tick more than one option where applicable.**   - Consult a doctor, including having a telehealth appointment - Ask a pharmacist for advice - Take medicines that you have at home which you bought without a prescription to help you feel better - Have extra rest - Take home remedies, such as sipping hot liquids - Take vitamins, such as vitamin C - Ask a family member or friend for advice - Search the internet to find advice on how to feel better - Take leftover or unused antibiotics - Visit the emergency department - Other, please specify |
| **2.2** | **Why would you decide to ask a pharmacist for advice? –** *if ‘ask a pharmacist for advice’ selected in Question 2.1*   - My symptoms are not serious enough to visit a doctor - Do not have time to wait for an appointment with a doctor - I am unable to get an appointment for a doctor - The pharmacist will tell me if I need to see a doctor - Easier to speak to a pharmacist for advice - I can get advice and purchase what I need to at the same time, if appropriate - Advice from the pharmacist is free - Pharmacist can issue me a medical certificate - Other |
|  | Please read the next section of the scenario.  **You decide to visit a pharmacy. While you do not feel your symptoms are serious, you want to feel better as soon as possible. You ask the pharmacist if you should book an appointment with a doctor to get antibiotics.**  **The pharmacist advises that they do not believe this is necessary based on your symptoms suggesting that you have a cold. They describe how antibiotics are not effective for treating viral infections, such as a cold.**  **The pharmacist advises that your cold should get better on its own after a week or so, and that rest will help. Understanding that your symptoms are bothering you, the pharmacist discusses over the counter medicines that may help and talks about how to use these products including any potential side effects.**  **The discussion includes what to do if you do not feel better over the next few days, including signs that you may need to seek more help from a doctor. The pharmacist also discusses having an influenza vaccination as you have not received one this year.** |
| **2.3** | **The following question will ask about the scenario you just read.**  **To what extent were the different pieces of information provided by the pharmacist in the scenario important for you to hear? Please rate using the scale from 1 to 5, where 1 is “very important” and 5 is “not at all important”.**   \|  \| 5- Not at all important \| 4 - Slightly important \| 3 - Important \| 2 - Fairly important \| 1 - Very important \| \| --- \| --- \| --- \| --- \| --- \| --- \| \| How antibiotics are not effective for viral infections (such as colds and flus) \|  \|  \|  \|  \|  \| \| Treatment options and advice to manage symptoms \|  \|  \|  \|  \|  \| \| Advice on when to visit a doctor if you do not feel better \|  \|  \|  \|  \|  \| \| Advice on preventing infections, such as vaccination \|  \|  \|  \|  \|  \| |
| **2.4** | **Have you visited a pharmacy for a cold and flu question in the last 6 months?**   - Yes - No – *Go to Section 3* |
| **2.5** | **Who were you asking the cold and flu question for?**   - Myself - Partner - Children - Other, please specify |
| **2.6** | **Using the scale below, please indicate your level of agreement on how accurate the scenario you read matches with your most recent experience with a pharmacist about a cold and flu enquiry.**   - Strongly disagree - Disagree - Neither agree nor disagree - Agree - Strongly agree |
| **SECTION 3 – ABOUT YOU** | |
| **3.1** | **With which gender do you identify most?**   - Male - Female - Non-binary or gender diverse - Prefer not to say |
| **3.2** | **What is your age?**   - [Free text] |
| **3.3** | **Which state or territory do you live in?**   - New South Wales - Victoria - Queensland - South Australia - Western Australia - Tasmania - Northern Territory - Australian Capital Territory |
| **3.4** | **Do you identify an Aboriginal and/or Torres Strait Islander person?**   - Yes, Aboriginal - Yes, Torres Strait Islander - Yes, Aboriginal and Torres Strait Islander - No - Prefer not to say |
| **3.5** | **How would you describe your cultural background?**   - English - Irish - Scottish - Chinese - Italian - German - Indian - Greek - Dutch - Australian - Other, please specify |
| **3.6** | **Apart from English, in which language(s) could you have a conversation about everyday things?**   - English only - Mandarin - Arabic - Cantonese - Vietnamese - Italian - Greek - Hindi - Spanish - Punjabi - Other, please specify |
|  | **END OF SURVEY** |
